# The crucial role of the TRPM7 kinase domain in the early stage of amelogenesis

**DOI:** 10.1038/s41598-017-18291-0

**Published:** 2017-12-22

**Authors:** Kayoko Ogata, Tomoyuki Tsumuraya, Kyoko Oka, Masashi Shin, Fujio Okamoto, Hiroshi Kajiya, Chiaki Katagiri, Masao Ozaki, Masayuki Matsushita, Koji Okabe

**Affiliations:** 1 Section of Cellular Physiology, Department of Physiological Sciences and Molecular Biology, Fukuoka Dental College, Fukuoka, Japan; 2 Section of Pediatric Dentistry, Department of Oral Growth and Development, Fukuoka Dental College, Fukuoka, Japan; 3 Department of Molecular and Cellular Physiology, Graduate School of Medicine, University of the Ryukyus, Okinawa, Japan

## Abstract

Transient receptor potential melastatin-7 (TRPM7) is a bi-functional protein containing a kinase domain fused to an ion channel. TRPM7 is highly expressed in ameloblasts during tooth development. Here we show that TRPM7 kinase-inactive knock-in mutant mice (TRPM7 KR mice) exhibited small enamel volume with opaque white-colored incisors. The TRPM7 channel function of ameloblast-lineage cells from TRPM7 KR mice was normal. Interestingly, phosphorylation of intracellular molecules including Smad1/5/9, p38 and cAMP response element binding protein (CREB) was inhibited in ameloblasts from TRPM7 KR mice at the pre-secretory stage. An immunoprecipitation assay showed that CREB was bound to TRPM7, suggesting that direct phosphorylation of CREB by TRPM7 was inhibited in ameloblast-lineage cells from TRPM7 KR mice. These results indicate that the function of the TRPM7 kinase domain plays an important role in ameloblast differentiation, independent of TRPM7 channel activity, via phosphorylation of CREB.

## Introduction

Tooth enamel, the hardest mineralized tissue in the body, is formed in several stages by ameloblasts that differentiate at each stage. During enamel formation, the cuboidal pre-ameloblasts of the pre-secretory stage change their morphology and differentiate into secretory ameloblasts with columnar shapes^1^. The ameloblasts of the secretory stage secrete components of the enamel matrix, including amelogenin, ameloblastin and enamelin, and the volume and thickness of the enamel are determined at this stage^2^. Next, during the maturation stage, the ameloblasts cycle between two phenotypes based on the morphology of the membrane facing the enamel surface: ruffle-ended ameloblasts and smooth-ended ameloblasts. The alteration of pH between ruffle-ended ameloblasts and smooth-ended ameloblasts is rhythmically regulated through cation transportation^3^. The entire process of enamel formation is orchestrated by complex signaling mechanisms that are highly regulated during each ameloblast stage; these mechanisms include growth factors, such as fibroblast growth factors, sonic hedgehog, bone morphogenetic proteins (BMPs) and Wnt proteins, as well as ion transportation^4–7^.

Transient receptor potential melastatin-7 (TRPM7), a member of the transient receptor potential (TRP) family, is a non-selective cation channel that displays outward rectification and is permeable to a number of divalent ions, including Mg^2+^ and Ca^2+^ ^8,9^. TRPM7 plays an essential role in the physiologic and biologic activity of many cell types. Some TRP channels, including TRPM7, are involved in sensory transduction in odontoblasts^10–12^, and activation of these channels by mechanical stimuli, stretch or temperature change is associated with pain^13–16^. However, little is known regarding the role of TRPM7 in enamel formation.

Interestingly, TRPM7 contains both an ion channel and a protein kinase in its carboxyl terminus and hence is a bi-functional protein (“chanzyme”) that can transport ions across the cell membrane as well as transduce signals to the cytosol^17,18^. Mice homozygous for a deletion of the TRPM7 kinase domain (*TRPM7* ^*ΔK/ΔK*^) show early lethality at embryonic day 7.5, whereas mice heterozygous for a deletion of the kinase domain (*TRPM7* ^*ΔK* /+^) survive to adulthood but exhibit reduced Mg^2+^ levels in plasma, bones and urine^19^. Recently, hypo-mineralization of teeth and cranial bones caused by a defect in alkaline phosphatase (ALPase) activity has been reported in *TRPM7* ^*ΔK* /+^ mice^20^. Because the mast cells derived from *TRPM7* ^*ΔK* /+^ mice demonstrated reduced TRPM7 currents^19^, Nakano and colleagues concluded that hypo-mineralization of teeth in *TRPM7* ^*ΔK* /+^ mice resulted from a defect in ion (Mg^2+^) transportation through the TRPM7 channel, the function of which had been affected by deletion of the kinase domain.

To address whether the kinase and ion channel functions of TRPM7 operate independently, we have generated TRPM7 kinase-inactive knock-in mutant mice that have normal TRPM7 ion channel function (TRPM7 KR mice). Our previous study reported that TRPM7 KR mice had normal serum levels of Mg^2+^ and Ca^2+^, showed normal growth during a 10-month follow-up period, and did not differ from wild-type (WT) mice with regard to body weight, food intake or locomotor activity^21^. Interestingly, TRPM7 KR mice exhibited defective enamel formation on the incisors. This phenotype led us to hypothesize that the kinase function of TRPM7 operates independently of the ion channel function in ameloblasts during enamel formation. In the present study, we report that the kinase domain of TRPM7 in ameloblasts functions independently of the TRPM7 ion channel to play a crucial role in ameloblast differentiation during the pre-secretory and secretory stages, possibly through phosphorylation of intracellular signaling molecules such as cAMP response element binding protein (CREB).

## Results

### TRPM7 kinase-inactive mutant mice display abnormalities of the enamel surface of the incisors

**Figure 1:**
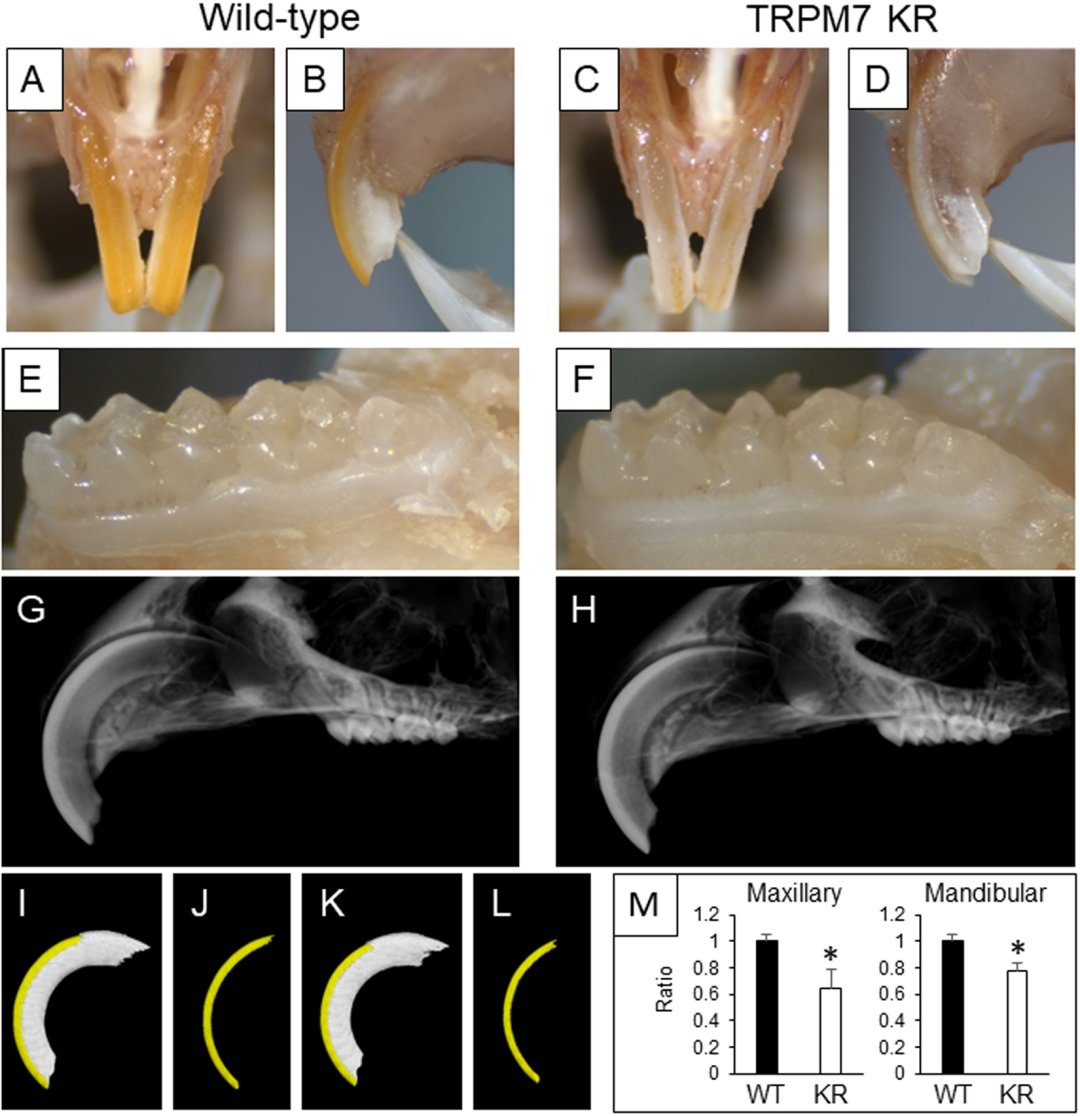
(**A**–**F**) Anterior (**A** and **C**) and lateral (**B** and **D**) views of the maxillary incisors and occlusal views of the mandibular molars (**E** and **F**) of 20-week-old wild-type (WT) (**A**,**B** and **E**) and TRPM7 KR (**C**,**D** and **F**) mice. (**G**,**H**) Sagittal views of the maxillary incisors of 16-week-old WT (**G**) and TRPM7 KR (**H**) mice, obtained using high-definition radiography. (**I**–**L**) 3D Reconstruction of upper incisor enamel (yellow) with/without dentin (white) from WT (**I**,**J**) and TRPM7 KR (**K**,**L**) mice using micro-CT analysis of (**G**) and (**H**), respectively. (**M**) The volume of the enamel of the maxillary and mandibular incisors of WT and TRPM7 KR mice aged 12–16 weeks, calculated from analysis of the micro-CT images. Data are presented as the mean ± standard deviation (*n* = 3). *P < 0.05. KR: TRPM7 KR mutant; WT: wild-type.

The appearance of the incisors in TRPM7 KR mice at 20 weeks of age is shown in Fig. 1. The maxillary incisors of wild-type (WT) mice had a smooth and yellowish surface, whereas those of TRPM7 KR mice lacked yellow pigmentation (Fig. 1A–D). On the other hand, the surface appearance and crown morphology of the molars were similar between WT and TRPM7 KR mice (Fig. 1E,F). Sagittal views of the maxillary incisors on high-definition radiographs did not reveal any clear differences between WT and TRPM7 KR mice (Fig. 1G,H). However, three-dimensional (3D) reconstructions of images obtained using micro-computed tomography (micro-CT) enabled the incisor enamel to be highlighted in yellow, allowing the enamel length and thickness to be determined (Fig. 1I–L). The volume of the enamel of both the upper and lower incisors was significantly smaller in TRPM7 KR mice than in WT mice (Fig. 1M).

### Maxillary incisors from TRPM7 KR mice show abnormal enamel prism structure and mineralization defects

**Figure 2:**
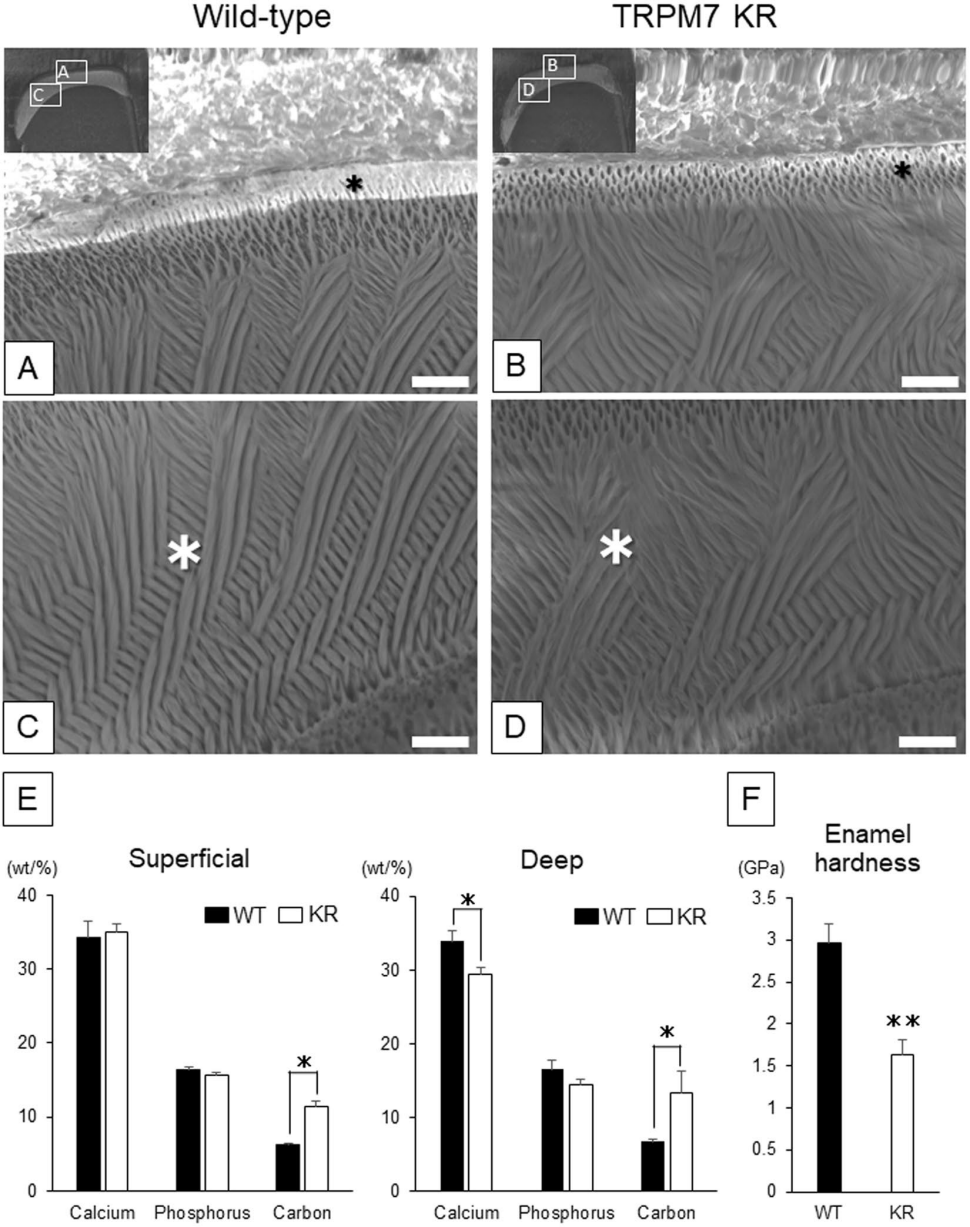
(**A**–**D**) Scanning electron microscopy (SEM) images showing the superficial (**A**,**B**) and deep (**C**,**D**) layers of the maxillary incisor enamel from 7-week-old wild-type (**A**,**C**) and TRPM7 KR (**B**,**D**) mice. The inset in each of (**A**) and (**B**) indicates the regions that were magnified to obtain (**A**,**C**) and (**B**,**D**), respectively. Scale bar: 10 μm (**A**–**D**). *Regions investigated using element mapping at the microstructural level. (**E**) The contents of calcium, phosphorus and carbon in the superficial and deep layers of incisor enamel from wild-type (WT) and TRPM7 KR mice. The content of each element is shown as the normalized concentration in weight percent (wt/%). (**F**) Measurements of enamel micro-hardness in WT and TRPM7 KR mice. The mean micro-hardness was 2.95 ± 0.24 GPa in WT mice and 1.64 ± 0.18 GPa in TRPM7 KR mice. Data in (**E**,**F**) are presented as the mean ± standard deviation (*n* = 3). KR: TRPM7 KR mutant; WT: wild-type. *P < 0.05, **P < 0.01.

Scanning electron microscopy (SEM) images of the enamel crystals of maxillary incisors from WT and TRPM7 KR mice are shown in Fig. 2. Cross-sectional images of incisor enamel demonstrated two layers, a superficial layer (Fig. 2A,B) and a deep layer (Fig. 2C,D). The density of the enamel rods in the superficial layer of the enamel was slightly lower in TRPM7 KR mice than in WT mice (Fig. 2A,B). In the deep layer of the enamel in WT mice, the rods were formed by tightly packed bundles of crystallites and inter-rod enamel was observed to have an enamel prism structure (Fig. 2C). However, in TRPM7 KR mice, the bundles of crystallites were loosely packed, and the enamel rods and inter-rod structures were slightly sparser than in WT mice (Fig. 2D). The contents of calcium, phosphorus and carbon in the superficial and deep enamel layers were examined by energy dispersive X-ray spectrometry (EDX) analysis. Compared with WT mice, TRPM7 KR mice exhibited a significantly lower calcium content in the deep enamel layer and a significantly higher carbon content in both the superficial and deep enamel layers (Fig. 2E). Furthermore, micro-hardness analysis revealed that the Vickers micro-hardness was significantly lower in incisors from TRPM7 KR mice than in those from WT mice (Fig. 2F).

### TRPM7 is highly expressed in teeth during their development

**Figure 3:**
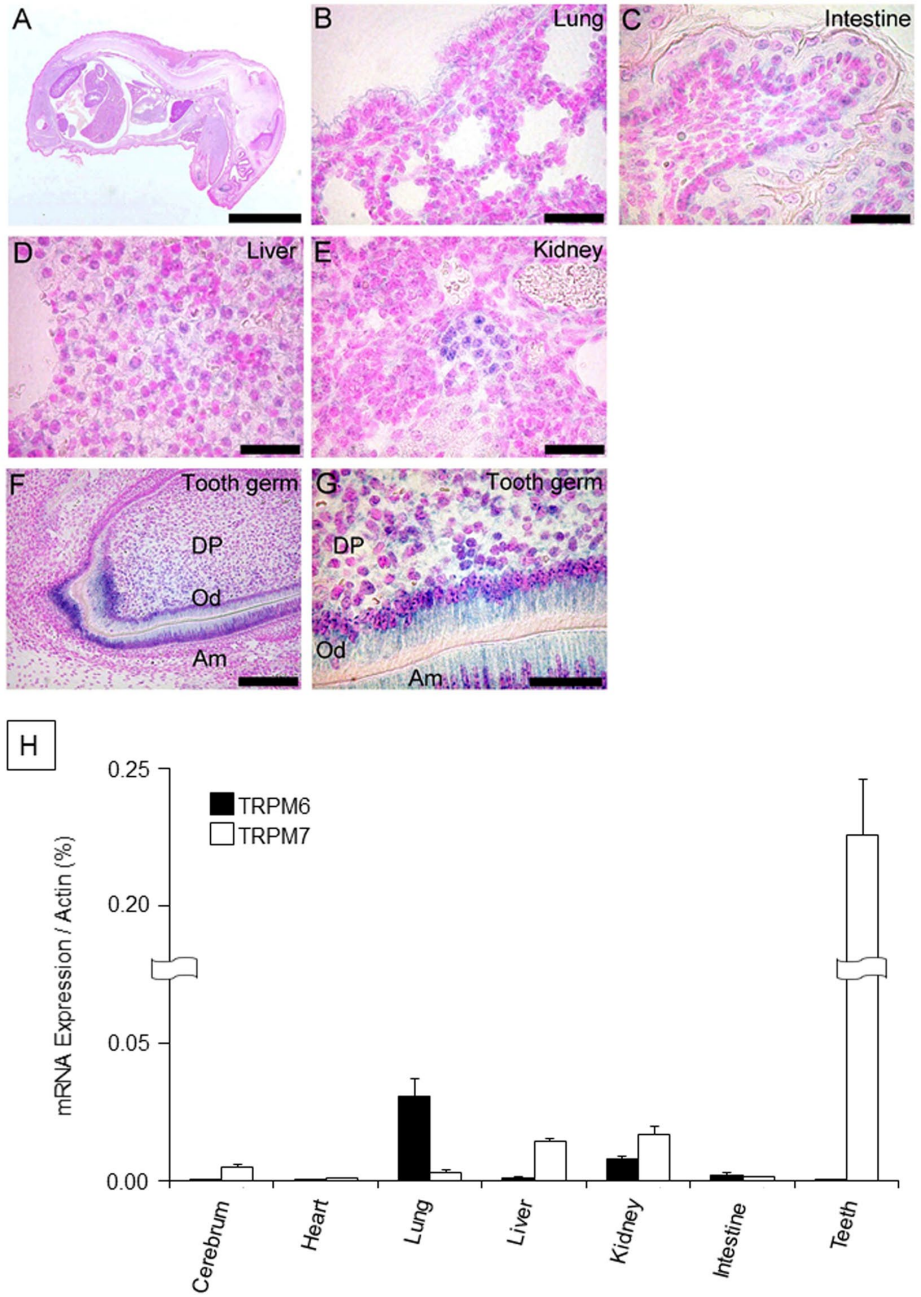
(**A**) The pattern of TRPM7 mRNA expression in a whole-body sagittal section at E18.5. (**B**–**G**) High-magnification images showing TRPM7 mRNA expression in the lung (**B**), intestine (**C**), liver (**D**), kidney (**E**) and tooth germ (**F**,**G**). (**H**) Quantitative RT-PCR analysis of TRPM7 and TRPM6 mRNA expression levels in various organs from 8-week-old wild-type mice. The mRNA expression levels of TRPM7 (white bars) and TRPM6 (black bars) are normalized to that of actin and presented as the mean ± standard deviation (*n* = 3). Am: ameloblast; DP: dental papilla; Od: odontoblast. Scale bars: 5 mm (**A**), 100 μm (**B**–**F**), 50 μm (**G**).

Since a remarkable phenotype was observed regarding the enamel of incisors from TRPM7 KR mice, we performed *in situ* hybridization using a whole-body sagittal section at E18.5 to detect TRPM7 mRNA expression in various tissues of WT mice. TRPM7 mRNA expression was clearly detected in the kidney but was weak in other tissues, including the lung, intestine and liver (Fig. 3A–E). Surprisingly, the expression levels of TRPM7 mRNA in the odontoblast and ameloblast layers of the maxillary incisors were notably higher than those in other tissues (Fig. 3F,G). Quantitative reverse-transcription polymerase chain reaction (qRT-PCR) experiments also demonstrated dramatically higher expression of TRPM7 mRNA in teeth compared with other tissues (Fig. 3H). qRT-PCR was used to observe TRPM6 mRNA expression, since TRPM6 is a close homologue to TRPM7 and also a bi-functional protein with a kinase domain. However, TRPM6 expression in teeth was very low (Fig. 3H). These observations suggest that TRPM7 is specifically required during tooth development.

### TRPM7 is expressed in odontoblasts and ameloblasts from the late bell stage

**Figure 4:**
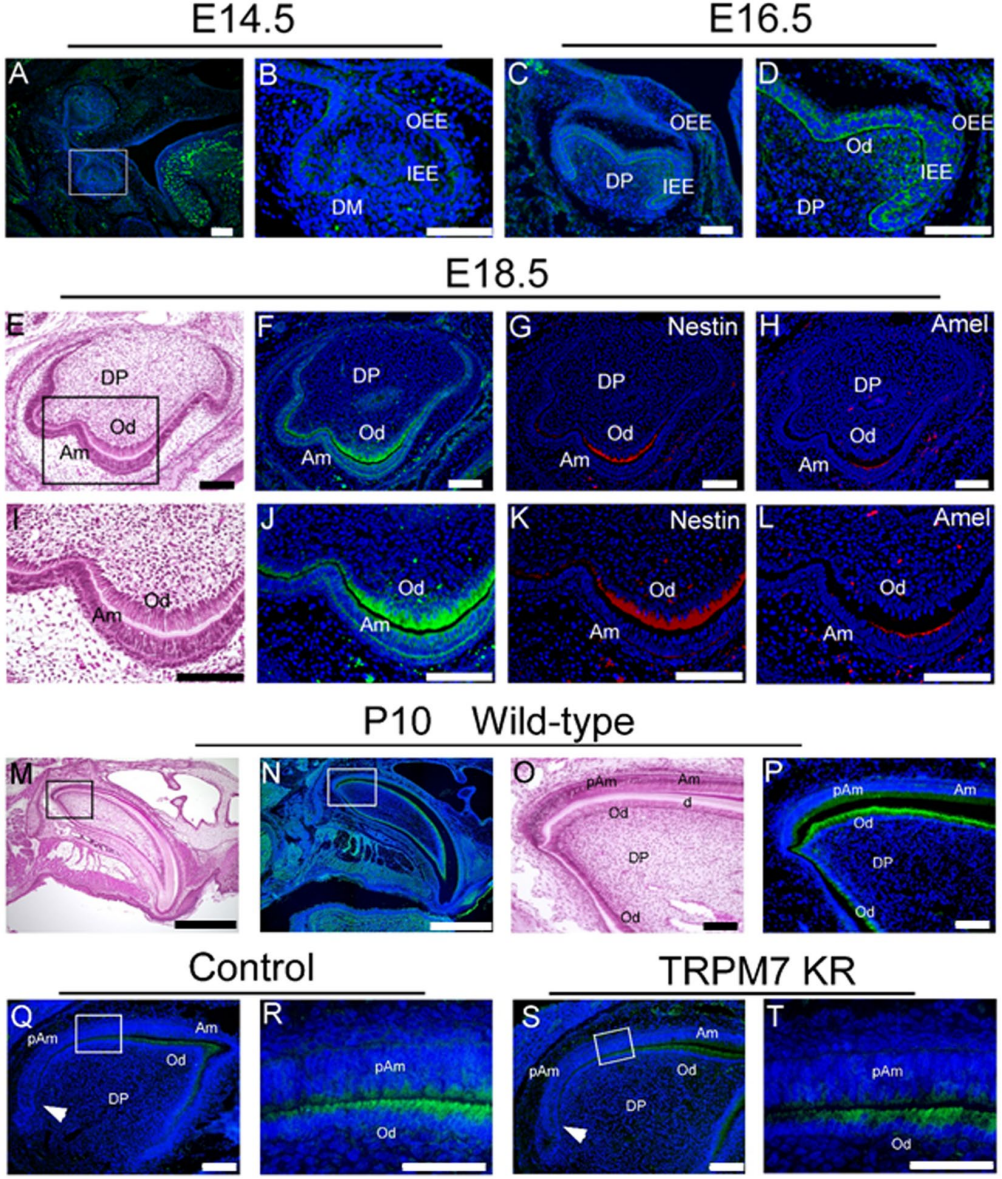
(**A**–**D**) TRPM7 protein expression in E14.5 (**A**,**B**) and E16.5 (**C**,**D**) molars, detected by immunohistochemistry. (**B**) and (**D**) are higher magnifications of the tooth germ shown in (**A**) and (**C**), respectively. (**E**–**L**) Adjacent sections of molars at E18.5 stained using different techniques. (**E**,**I**) Hematoxylin and eosin staining. (**F**,**J**) TRPM7 protein expression detected by immunohistochemistry. (**G**,**K**) Expression of nestin protein (a marker of differentiated odontoblasts) detected by immunohistochemistry. (**H**,**L**) Expression of amelogenin protein (a marker of differentiated ameloblasts) detected by immunohistochemistry. (**M**) Hematoxylin and eosin staining of an incisor from a wild-type mouse at P10. (**N**) TRPM7 protein expression, detected by immunohistochemistry, in an incisor from a wild-type mouse at P10. (**O**) Higher magnification of the box in (**M**). (**P**) Higher magnification of the box in (**N**). (**Q**) TRPM7 protein expression, detected by immunohistochemistry, in an incisor from a control (heterozygous) mouse. (**R**) Higher magnification of the box in (**Q**), showing pre-ameloblasts and odontoblasts. (**S**) TRPM7 protein expression, detected by immunohistochemistry, in an incisor from a TRPM7 KR mouse. (**T**) Higher magnification of the box in (**S**), illustrating the pre-ameloblasts and odontoblasts. White arrowheads indicate the cervical region. Scale bars: 100 μm (**A**–**Q**, **S**), 50 μm (**R**, **T**). Am: ameloblast, d: dentin matrix, DM: dental mesenchyme, DP: dental papilla; IEE: inner enamel epithelium; Od: odontoblast; OEE: outer enamel epithelium; pAm: pre-ameloblast.

In view of the high expression of TRPM7 mRNA observed in teeth, we carried out immunohistochemistry experiments to analyze the protein expression of TRPM7 during tooth development. At E14.5, TRPM7 expression was barely detectable in bud-stage tooth germ (Fig. 4A,B). At E16.5, a weak signal was detected in the inner and outer enamel epithelium and in the odontoblast layer of the dental papilla (Fig. 4C,D). At E18.5, a thin layer of dentin was seen in the cusp region of the tooth germ at the crown-forming stage (Fig. 4E,I), and TRPM7 expression was clearly evident in the odontoblast and ameloblast layers in the cusp region (Fig. 4F,J). To gain more insight into the distribution of TRPM7 protein expression, differentiated odontoblasts and ameloblasts were labeled with nestin and amelogenin, respectively (Fig. 4G,H,K,L). The regions showing TRPM7 expression were slightly wider than those showing nestin and amelogenin expression (Fig. 4F,J), suggesting that TRPM7 was expressed from the pre-odontoblast and pre-ameloblast stages.

Next, to examine the expression of TRPM7 at each stage of ameloblast differentiation, sagittal sections of the upper incisor at P10 were prepared. In WT mice, TRPM7 was observed in all differentiating and differentiated ameloblasts from the pre-secretory stage onward (Fig. 4M–P). At the initiating stage of enamel formation, TRPM7 was not detected in the cervical region of the ameloblast layer (Fig. 4Q, arrowhead). Based on the observed pattern of TRPM7 expression in the incisor during enamel formation, it appears that TRPM7 is expressed continuously in the inner enamel epithelium from the pre-secretory stage onward (Fig. 4R). We also examined the expression of TRPM7 in the upper incisor of TRPM7 KR mice. Immunohistochemical staining of TRPM7 was similarly detected in control (heterozygous) and TRPM7 KR mice (Fig. 4S,T).

### Inactivation of TRPM7 kinase activity does not alter TRPM7 channel function in inner enamel epithelial cells

**Figure 5:**
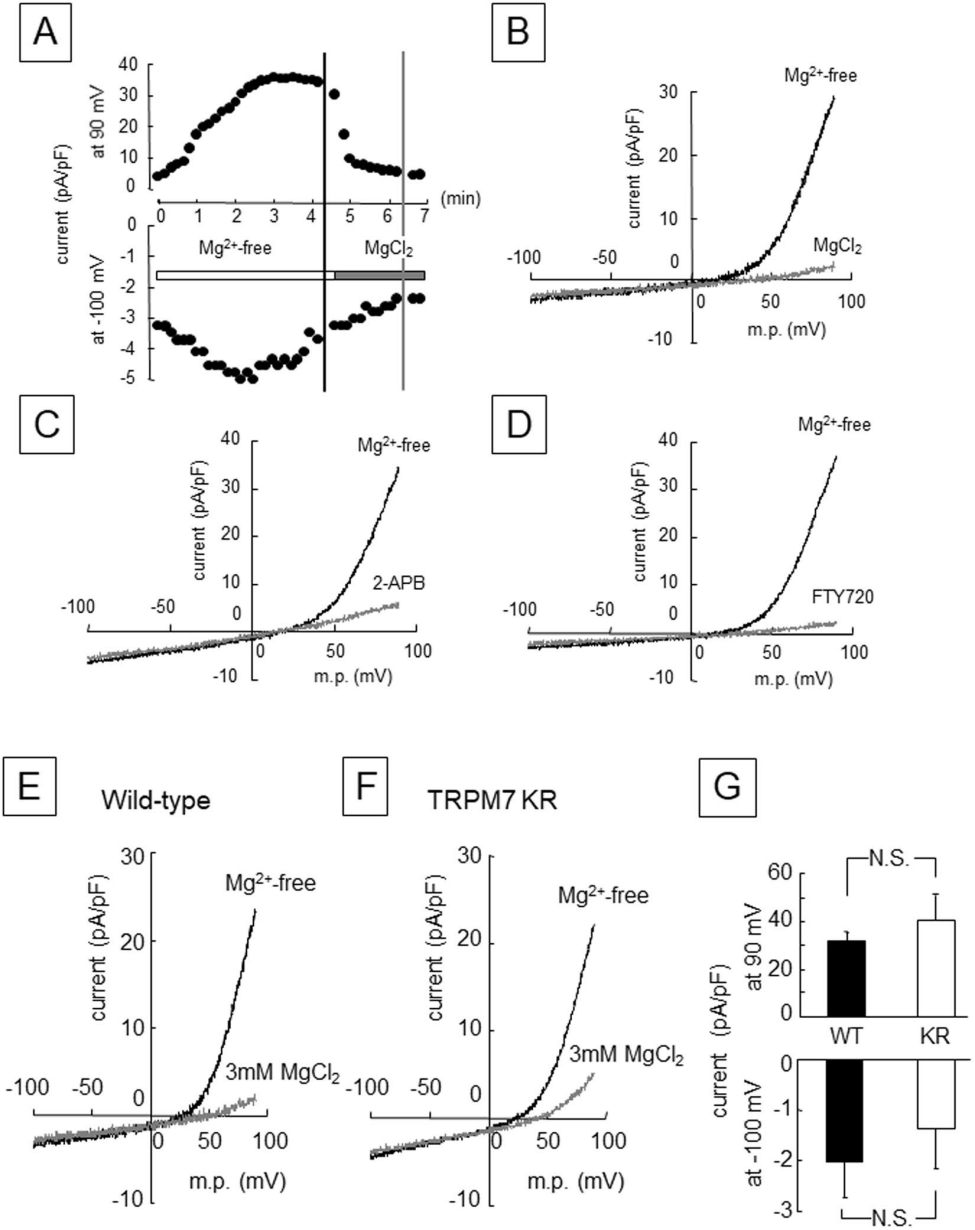
(**A**–**D**) Mg^2+^-inhibited cation (MIC) currents recorded from inner enamel epithelium-lineage cells from wild-type mice. (**A**) Time course of the change in membrane current at +90 mV and −100 mV during intracellular and extracellular Mg^2+^ depletion and following the application of 3 mM MgCl_2_ to the extracellular solution. (**B**) Current-voltage (*I-V*) relationships obtained at the time points indicated by the vertical bars in (**A**), i.e. before (black line) and after (gray line) application of 3 mM MgCl_2_. (**C**) *I-V* relationships obtained before (black line) and after (gray line) application of 2-aminoethoxyphenylborate (2-APB; 500 μM), a non-specific modulator of several TRP channels including TRPM7. (**D**) *I-V* relationships obtained before (black line) and after (gray line) application of FTY720 (10 μM), also an inhibitor of TRPM7 channel activity. (**E**,**F**) *I-V* relationships recorded from inner enamel epithelium-lineage cells from wild-type and TRPM7 KR mutant mice. The *I-V* relationships were obtained before (Mg^2+^-free; black line) and after (gray line) application of 3 mM MgCl_2_. (**G**) The graph on the right compares the amplitudes of the MIC currents (net current inhibited by 3 mM MgCl_2_) at +90 mV and −100 mV. Data are expressed as the mean ± standard error of the mean (*n* = 4). KR: TRPM7 KR mutant; m.p.: membrane potential; N.S.: not significant (*P* ≥ 0.05); WT: wild-type.

The immunohistochemistry experiments confirmed that TRPM7 was abundantly expressed in ameloblasts during tooth development (Fig. 4). Therefore, we used the whole-cell configuration of the patch-clamp technique to examine the ion channel activity of TRPM7 in inner enamel epithelium-lineage cells from WT mice (Fig. 5). Removal of extracellular and intracellular Mg^2+^ (the latter by dialyzing the cell with Mg^2+^-free patch pipette solution) potentiated the magnitudes of cation currents recorded at holding potentials of +90 and −100 mV; the outward conductance was substantial whereas the inward conductance was much smaller. The addition of 3 mM MgCl_2_ to the extracellular solution reduced the magnitudes of both currents (Fig. 5A). The current-voltage (*I-V*) relationship of the whole-cell current in the absence of Mg^2+^ (Mg^2+^-free) showed steep outward rectification with small inward currents, and the outward currents were substantially reduced by subsequent external application of Mg^2+^ (3 mM MgCl_2_) (Fig. 5B). These observations are consistent with the presence of a Mg^2+^-inhibited cation (MIC) current in inner enamel epithelium-lineage cells, which likely represents the activity of TRPM7 channels^22^. Moreover, the MIC current was inhibited by extracellular application of 2-aminoethoxyphenylborate (2-APB), a non-specific modulator of several TRP channels (Fig. 5C). External application of 2-amino-2-[2-(4-octylphenyl)ethyl]-1,3-propanediol hydrochloride (FTY720), a potent inhibitor of TRPM7 channel activity^23^, also blocked the MIC current (Fig. 5D). These findings suggest that TRPM7 functions as a cation channel in ameloblastic cells during enamel formation. Next, we compared the amplitudes of the MIC currents recorded from inner enamel epithelium-lineage cells obtained from WT and TRPM7 KR mice (Fig. 5E–G). The *I-V* relationships obtained from both WT and TRPM7 KR mutant cells under Mg^2+^-free conditions showed steep outward rectification. Subsequent application of Mg^2+^ (3 mM MgCl_2_) to the bathing solution reduced the amplitudes of the currents in both WT and TRPM7 KR mutant cells, suggesting the presence of a MIC current in TRPM7 KR mutant cells that was similar to that in WT cells (Fig. 5E,F). We further confirmed that the MIC currents in TRPM7 KR mutant cells were abolished by extracellular application of 2-APB or FTY720 (data not shown). There were no significant differences between WT and TRPM7 KR mutant cells with regard to the amplitude of the outward component of the MIC current at +90 mV or the amplitude of the small inward component at −100 mV (Fig. 5G). These results suggest that TRPM7 kinase activity is not essential for TRPM7 channel activity.

### TRPM7 KR mice show reduced phosphorylation of intracellular signaling proteins in ameloblasts at the pre-secretory and secretory stages

**Figure 6:**
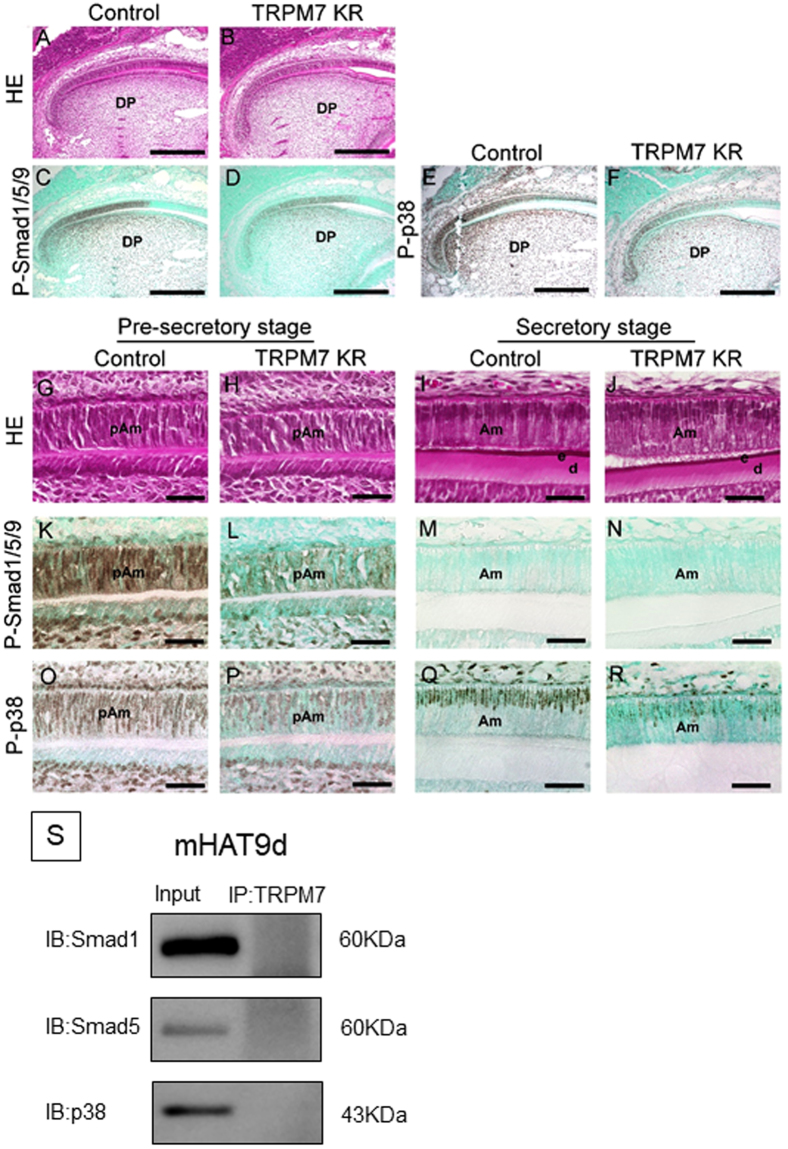
(**A**,**B**) Hematoxylin and eosin staining of incisors from control (heterozygous) (**A**) and TRPM7 KR (**B**) mice at P10. (**C**,**D**) Immunohistochemical detection of the expression of P-Smad1/5/9 in incisors from control (**C**) and TRPM7 KR (**D**) mice at P10. (**E**,**F**) Immunohistochemical detection of the expression of P-p38 in incisors from control (**E**) and TRPM7 KR (**F**) mice at P10. (**G**–**J**) Higher magnification views of regions in (**A**) and (**B**) to show the morphology of the incisors from control (**G**,**I**) and TRPM7 KR (**H**,**J**) mice at the pre-secretory (**G**,**H**) and secretory (**I**,**J**) stages. (**K**–**N**) Immunohistochemical detection of P-Smad1/5/9 expression in incisors from control (**K**,**M**) and TRPM7 KR (**L**,**N**) mice at the pre-secretory (**K**,**L**) and secretory (**M**,**N**) stages. (**O**–**R**) Immunohistochemical detection of P-p38 expression in incisors from control (**O**,**Q**) and TRPM7 KR (**P**,**R**) mice at the pre-secretory (**O**,**P**) and secretory (**Q**,**R**) stages. (**S**) Immunoblotting analysis of extracts from mHAT9d cells, using anti-Smad1, anti-Smad5 or anti-p38 antibodies after immunoprecipitation by anti-TRPM7 antibody. Input: total cell lysate; IP: immunoprecipitation. Full-length blots are presented in Supplementary Figure 1. Am: ameloblast; d: dentin matrix; DP: dental papilla; e: enamel matrix; HE: hematoxylin and eosin; pAm: pre-ameloblast. Scale bars: 100 μm (**A**–**F**), 50 μm (**G**–**R**).

Next, we investigated intracellular signaling mechanisms in incisor ameloblasts to gain more insight into the kinase function of TRPM7. In histologic samples of incisors stained with hematoxylin-eosin, the morphology of the ameloblasts at the pre-secretory and secretory stages was similar between control (heterozygous) and TRPM7 KR mice (Fig. 6A,B,G–J). We then studied the expression of Smad1/5/9 and p38, which are intracellular transcription factors recruited by BMP, a critical regulator of ameloblast differentiation. In control mice, phospho-Smad1/5/9 (P-Smad1/5/9) was continuously expressed in ameloblasts at the pre-secretory stage but was not detected at the secretory stage (Fig. 6C,K,M). A similar expression pattern was also observed in TRPM7 KR mice, but the expression of P-Smad1/5/9 at the pre-secretory stage was notably lower (Fig. 6D,L,N). In control mice, phospho-p38 (P-p38) was expressed throughout the entire inner enamel epithelium (Fig. 6E). P-p38 expression was evident at the pre-secretory stage and detected in the nuclei at the secretory stage (Fig. 6O,Q). However, in TRPM7 KR mice, P-p38 expression was slightly lower at the pre-secretory stage (Fig. 6P) and was minimal at the secretory stage (Fig. 6R). During maturation, the expression of P-Smad1/5/9 in WT was not observed in ameloblasts. In addition, the immunoreaction of P-p38 antibody was weakly observed in WT, and little was seen in TRPM7 KR mice (data not shown).

The data described above imply that ameloblasts in TRPM7 KR mice show a downregulation of Smad signaling and mitogen-activated protein kinase (MAPK) signaling (two pathways recruited by BMP) at both the pre-secretory and secretory stages. To establish whether the kinase domain of TRPM7 could directly phosphorylate these signaling molecules, we performed an immunoblotting assay using mHAT9d cells, which are a dental epithelial cell line derived from the apical bud of a mouse incisor. However, binding between TRPM7 and Smad1, Smad5 or p38 was not detected (Fig. 6S).

### The TRPM7 kinase domain directly phosphorylates CREB in the ameloblast during enamel formation

**Figure 7:**
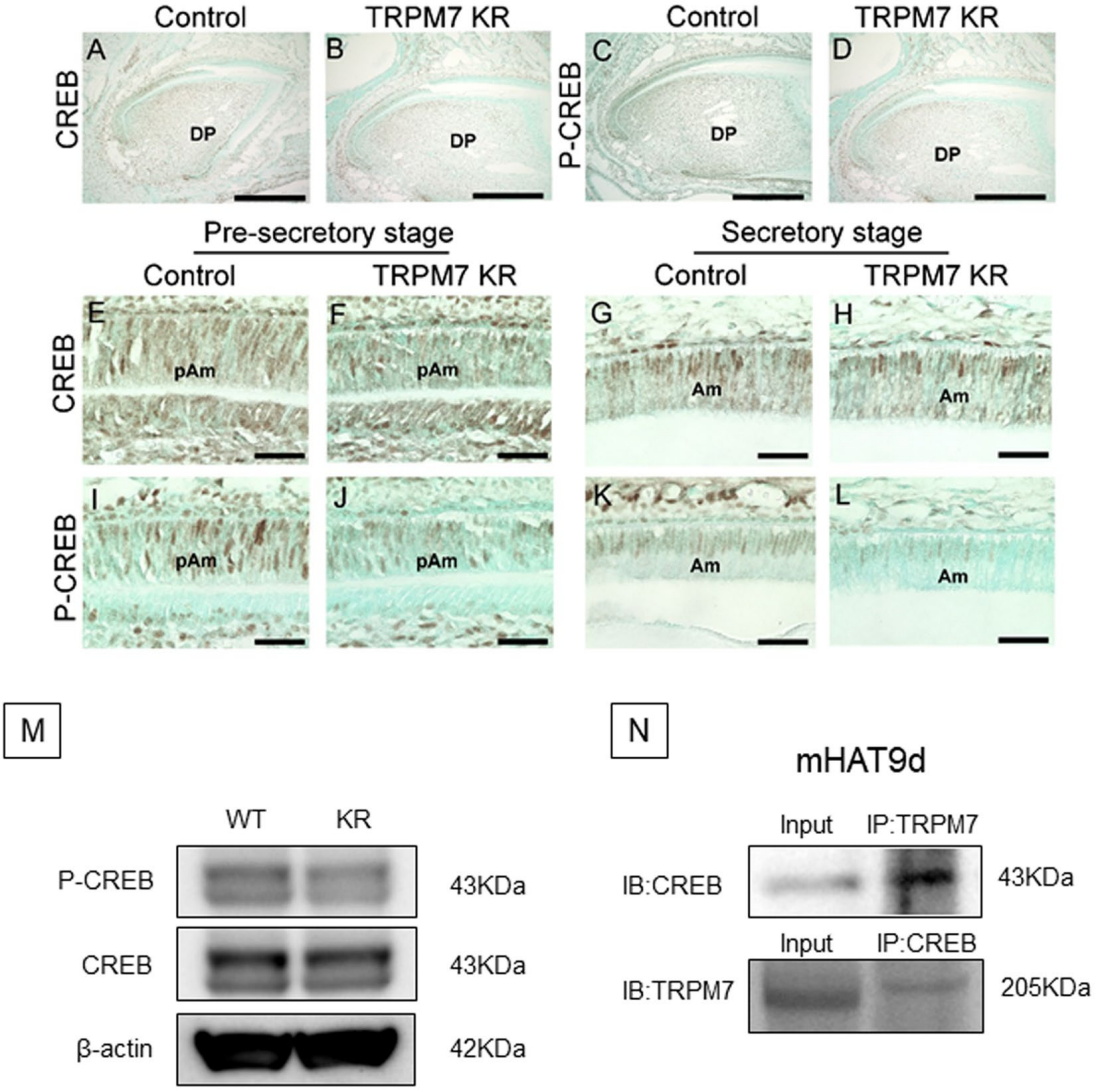
(**A**–**D**) Immunohistochemical detection of the expressions of CREB (**A**,**B**) and P-CREB (**C**,**D**) in incisors from control (heterozygous) (**A**,**C**) and TRPM7 KR (**B**,**D**) mice at P10. (**E**–**L**) Higher magnification views of regions in (**A**–**D**) to show the expression of CREB (**E**–**H**) and P-CREB (**I**–**L**) in incisors from control (**E**,**G**,**I**,**K**) and TRPM7 KR (**F**,**H**,**J**,**L**) mice at the pre-secretory stage (**E**,**F**,**I**,**J**) and secretory stage (**G**,**H**,**K**,**L**). (**M**) Western blot analysis of CREB and P-CREB expression in first molar tooth germ at P2. Full-length blots are presented in Supplementary Figure 2. (**N**) Immunoblotting analysis of extracts from mHAT9d cells, using anti-CREB antibody after immunoprecipitation by anti-TRPM7 antibody (upper lane) or anti-TRPM7 antibody after immunoprecipitation by anti-CREB antibody (lower lane). Input: total cell lysate; IP: immunoprecipitation. Full-length blots are presented in Supplementary Figure 3. Am: ameloblast; d: dentin matrix; DP: dental papilla; e: enamel matrix; KR: TRPM7 KR mutant; pAm: pre-ameloblast; WT: wild-type. Scale bars: 100 μm (**A**–**D**), 50 μm (**E**–**L**).

It was recently reported that the TRPM7 kinase domain is able to directly phosphorylate CREB peptide and full-length CREB in breast cancer cells^24^. Therefore, we examined the expression of CREB and phosphorylated CREB (P-CREB) in incisors from control (heterozygous) and TRPM7 KR mice (Fig. 7A–D). CREB expression was observed in ameloblasts at both the pre-secretory and secretory stages and was comparable between TRPM7 KR and control mice (Fig. 7E–H). However, P-CREB levels were reduced in TRPM7 KR mice at the pre-secretory and secretory stages (Fig. 7I–L). Western blot analysis also showed lower levels of phosphorylated CREB in TRPM7 KR mice compared with WT mice, whereas CREB protein expression was comparable between TRPM7 KR and WT mice (Fig. 7M). Interestingly, immunoblotting experiments detected the binding of CREB to TRPM7 (Fig. 7N). Based on these data, we suggest that TRPM7 kinase activity influences the early stage of enamel formation by phosphorylating CREB in ameloblasts at the pre-secretory and secretory stages.

## Discussion

There has been recent interest in the biologic function of ion channels and transporters during tooth development. Several types of ion channel and transporter have been identified as having important roles during amelogenesis^25,26^. In the present study, we focused on TRPM7, which is bi-functional in that it can act as an ion channel and a protein kinase. The kinase domain of TRPM7 belongs to an atypical alpha-kinase family, but its target substrates *in vivo* have yet to be identified. Mice with deletion of the TRPM7 kinase domain (*TRPM7* ^*ΔK*^) have been generated with the aim of improving our understanding of the function of this domain^19^. Homozygous *TRPM7* ^*ΔK/ΔK*^ mice showed early lethality, whereas heterozygous mutants survived but developed hypomagnesaemia, suggesting that deletion of the kinase domain resulted in dysfunction of the TRPM7 channel domain. Heterozygous *TRPM7* ^*ΔK*/+^ mice exhibited severe hypo-mineralization of the craniofacial structure, including the incisors, molars and cranial bones, that was caused by a low ALPase activity^20^. Impaired ALPase activity in *TRPM7* ^*ΔK*/+^ mice was partially rescued by pre-incubation with a solution containing a high concentration of Mg^2+^, suggesting that the hypo-mineralization of enamel in *TRPM7* ^*ΔK*/+^ mice was caused by a defect in TRPM7 channel function that accompanied deletion of the kinase domain. However, a definitive conclusion regarding the role of TRPM7 kinase in tooth development could not be drawn because it was not directly demonstrated whether ameloblasts from *TRPM7* ^*ΔK*/+^ mice showed a defect in TRPM7 current^20^.

In the present study, we demonstrated the existence of a TRPM7 current in inner enamel epithelium-lineage cells and showed that a defect in TRPM7 kinase activity did not affect TRPM7 channel function in inner enamel epithelium-lineage cells from TRPM7 KR mice. Kinase-independent TRPM7 channel activity has also been reported in peritoneal macrophages isolated from TRPM7 KR mice^21^. In our experiments, the TRPM7 current in inner enamel epithelium-lineage cells was similar between TRPM7 KR and WT mice in terms of its amplitude and sensitivity to inhibitors, indicating that our TRPM7 KR mice are well suited to studies of the role of TRPM7 kinase in tooth formation.

Although abnormalities of the enamel tip are generally observed with defects in tooth mineralization, no such abnormalities were evident in macroscopic views of the incisors from TRPM7 KR mice. However, SEM, EDX and micro-hardness analyses clearly showed that incisors from TRPM7 KR mice contained hypoplastic enamel with a disturbed structure. Therefore, we speculate that the small volume of enamel in TRPM7 KR mice revealed by micro-CT analysis resulted from tooth wear by mastication. Furthermore, analysis of the organic component of the enamel in incisors from TRPM7 KR mice suggests that there may be dysfunction of enamel matrix degradation. Thus, we examined amelogenesis in the incisor because this allowed observation of the entire process of ameloblast differentiation.

BMP signaling is recognized as a critical regulator of enamel formation. Conditional deletion of BMP2 and BMP4 using K14-cre mice (which have keratin-14 promoter-driven cre recombinase) resulted in amelogenesis imperfecta caused by downregulation of the removal of enamel matrix protein during maturation^27^. Spatiotemporal variations in the secretion of BMPs in dental epithelium occur during tooth development^28^. BMP4 and BMP7 are expressed in the pre-ameloblast layer of the mouse incisor, and these BMPs induce the expression of p21, a cell cycle inhibitor. BMP4 is intensely expressed in the dental mesenchyme that lies adjacent to differentiating epithelial cells and hence is regarded as a major regulator of ameloblast differentiation^28^. Generally, BMPs signal through two main pathways: Smad transcription factors (canonical) and MAPK pathways (non-canonical) that include extracellular signal-regulated kinase (Erk), c-Jun N-terminal kinases (JNKs) and p38 kinases^29,30^. A previous study demonstrated that Smad4 and p38 MAPK are functionally redundant during the early stage of enamel formation^31^, indicating that both Smad-dependent and p38-dependent pathways contribute to the mechanisms underlying enamel formation. Conditional deletion of Smad4 in the dental epithelium of K14-cre mice resulted in defective molar cusp formation, but the phenotype of the incisor could not be observed due to embryonic lethality^31^. Conditional deletion of p38α in K14-cre mice (*p38α* ^K14^) led to impaired patterning of the dental cusps and severe defects of enamel formation in the incisors^32,33^. Furthermore, *p38α* ^K14^ mice showed excessive proliferation of ameloblasts during the secretory stage, suggesting that non-canonical BMP signaling plays a role mainly during the secretory stage.

In the present study, the ameloblasts of TRPM7 KR mice showed downregulation of both canonical and non-canonical BMP signaling pathways at the pre-secretory and secretory stages. However, binding between TRPM7 and Smad1/Smad5 or p38 was not detected by immunoprecipitation, indicating that the downregulation of BMP signaling in the ameloblasts of TRPM7 KR mice was secondary to mutation of the kinase domain.

CREB is ubiquitously expressed in mouse tissues and is regarded as the predominant member of the CREB/ATF family in many cell types^34^. No previous studies have examined the role of CREB phosphorylation in ameloblasts. However, CREB-binding protein has been identified in the nuclei of rat incisor ameloblasts^35^, and mRNA expression of the OASIS gene (which encodes a CREB/ATF family member) has been detected in mouse inner enamel epithelium during the cap and bell stages (E14.5–E18.5)^36^. The TRPM7 kinase domain has been reported to phosphorylate CREB peptide in breast cancer cells^24^. Based on this previous report, we performed an immunohistochemistry analysis and found that CREB was expressed in ameloblasts at the pre-secretory and secretory stages of enamel formation. Although CREB expression was comparable between control and TRPM7 KR mice, the expression of phosphorylated CREB was lower in ameloblasts from TRPM7 KR mice at the pre-secretory and secretory stages. Furthermore, the binding of CREB and TRPM7 in ameloblast cells was detected by an immunoprecipitation assay. Therefore, we suggest that the TRPM7 kinase domain plays an important role in the phosphorylation of CREB at the pre-secretory and secretory stages and that this interaction subsequently affects the process of ameloblast maturation. It may be that, due to continuous growth of the incisors, defective enamel formation was more readily visualized in the incisors of TRPM7 KR mice than in the molars. It is also possible that other, complementary, signaling pathways may compensate for deficient CREB phosphorylation in the molars of TRPM7 KR mice, thereby allowing the formation of enamel to proceed normally.

We further hypothesize that inhibition of CREB phosphorylation may in turn lead to downregulation of BMP signaling in ameloblasts from TRPM7 KR mice. An interaction between CREB and BMP signaling pathways has been demonstrated previously, with phosphorylated-Smad1/5/8, phosphorylated-CREB, and CREB-binding protein reported to form a transcriptional complex in C2C12 cells^37^. It is possible that defective phosphorylation of CREB affects the phosphorylation of Smad1/5/9. The observation of downregulated BMP signaling in ameloblasts from TRPM7 KR mice during the early stage may be associated with reduced phosphorylation of CREB by the TRPM7 kinase domain.

Ameloblasts in the pre-secretory and secretory stages show a rapid cytoskeletal reorganization to prepare for secretion of enamel matrix, with the formation of Tomes’ processes and the development of mitochondria and Golgi apparatus in the cytoplasm. Therefore, these stages require the expression of ATP-dependent ion channels and transporters^5^. The present study has shown that, among the various ion channels and transporters present in ameloblasts, TRPM7 has a specific role in enamel formation that is mediated by the function of its kinase domain.

The immunohistochemistry experiments demonstrated that TRPM7 was expressed in odontoblasts as well as in ameloblasts. Furthermore, P-CREB was also expressed in odontoblasts from control mice and was downregulated in TRPM7 KR mice. TRPM7 expressed in odontoblasts has been suggested to play a role in mineralization during dentin formation^10^. Furthermore, TRPM7 has been proposed to be involved in the process of dental pulp repair through regulation of the proliferation, migration and osteogenic differentiation of human dental pulp stem cells^38^. In view of these observations, we have performed SEM experiments to compare dentin formation between TRMP7 KR mice and control mice, and we have also carried out an odontoblast marker analysis. However, these experiments did not detect any notable differences between TRPM7 KR mice and control mice (data not shown). We speculate that the ion channel function of TRPM7 may be more important than the kinase function for dentin formation. Furthermore, in tissues other than the enamel, alternative pathways may be capable of fully compensating for a deficiency in TRPM7 kinase activity and reduction in CREB phosphorylation level.

Nonetheless, the function of TRPM7 during enamel formation, including the role of its ion channel, remains incompletely understood. Thus, further investigations are required using conditional knockout techniques to avoid the early embryonic lethality seen in conventional TRPM7 knockout mice.

In conclusion, the data presented in this study indicate that the kinase domain of TRPM7 functions independently of the ion channel during the early stages of ameloblast differentiation and has a crucial role in the activation of BMP signaling through phosphorylation of CREB.

## Methods

The Animal Care Committee of Fukuoka Dental College approved all animal procedures used in this study. The protocol for these experiments was reviewed and approved by the Animal Committee and Research Ethics Committee of Fukuoka Dental College (no. 15005, 15009). The experimental methods were performed according to the regulations and guidelines established by the Animal Committee and Research Ethics Committee of Fukuoka Dental College.

### TRPM7 KR knock-in mice

TRPM7 kinase-inactive mutant mice (TRPM7 KR mice) had been generated previously. Exon 33 of *Trpm7* was point-mutated to change the lysine residue at position 1646 to arginine (K1646R). Homozygous TRPM7 KR mice exhibit normal growth, locomotor activity and serum Mg^2+^ and Ca^2+^ levels^21^.

### Micro-CT analysis

The jaws of mice aged 12–16 weeks were dissected out and fixed with ethanol. Samples were imaged with a SkyScan 1176 X-ray micro-CT system (Bruker BioSpin, Rheinstetten, Germany). The operating settings for the X-ray source were set to 50 kV and 500 µA, and image reconstitution was carried out using NRecon software (Bruker microCT, Kontich, Belgium). Following reconstruction, 3D image processing and analysis were carried out using DataViewer and CTAn software (Bruker microCT). The enamel volume for WT mice was defined as 1.0, and relative amounts for TRPM7 KR mice were calculated accordingly.

### SEM/EDX analysis

The maxillary bone and incisors of WT and TRPM7 KR mice aged 7 weeks were dissected out and fixed with ethanol. To observe the enamel crystals, the specimens were embedded in epoxy resin (Oken Epok, Okenshoji Co., Ltd., Tokyo, Japan) and cut using a diamond disk. The tissues were treated with 40% phosphoric acid for 10 s and 10% sodium hypochlorite for 30 sec. The specimens were imaged with a variable pressure scanning electron microscope (Miniscope TM3000, Hitachi, Tokyo, Japan). Element mapping at the microstructural level was carried out using an EDX spectrometry system (Quantax70, Bruker, Kanagawa, Japan).

### Vickers micro-hardness testing

Right mandibles were dissected from WT and TRPM7 KR mice aged 7 weeks, washed and dehydrated with graded ethanol. The samples, which were kept moist at all times, were fixed by composite resin on the metal plate and sagittally ground. (Handimet 2 Roll Grinder, Buehler, Lake Bluff, IL, USA) and automatic lapping machine (ml-160a, Maruto Instrument Co., Ltd., Tokyo, Japan). Incisor enamel micro-hardness was measured using a Vickers micro-hardness tester (MXT50, Matsuzawa, Co., Ltd, Akita, Japan) under a 100-g load for 10 sec.

### *In situ* hybridization

*In situ* hybridization experiments followed standard procedures. Samples fixed in 4% paraformaldehyde (PFA) were dehydrated by passage through a graded ethanol series and embedded in paraffin. Serial tissue sections were treated with proteinase K for 15 min at room temperature. TRPM7 riboprobes were generated by *in vitro* transcription using digoxigenin-labeled UTP, in accordance with the manufacturer’s instructions (Roche Diagnostics Corp., Indianapolis, IN, USA). Several negative controls (e.g. sense probe and no probe) were run in parallel with the experimental reaction.

### Immunohistochemical analysis

The heads of C57BL6 (WT), homozygous (TRPM7 KR) and heterozygous (Control) mice were dissected out and fixed in 10% neutral buffered formalin solution or 4% PFA overnight. Tissues were decalcified with Morse’s solution for 3 days and embedded in paraffin. Sections (4–7 μm thick) were cut using a microtome (RM2125 RTS; Leica Microsystems, Tokyo, Japan). The primary antibodies used were: rabbit polyclonal anti-TRPM7 (Abcam, Cambridge, UK), chicken polyclonal anti-nestin (Novus Biological, Littleton, CO, USA), rabbit polyclonal anti-amelogenin (Santa Cruz Biotechnology, Santa Cruz, CA, USA), rabbit monoclonal anti-phospho-Smad1/5/9 (Cell Signaling Technology, Danvers, MA, USA), rabbit polyclonal anti-phosho-p38 MAPK (Cell Signaling Technology), and rabbit monoclonal anti-phospho-CREB (Ser133) and anti-CREB (Cell Signaling Technology) antibodies. The secondary antibodies used were: Alexa Fluor® 488 anti-rabbit immunoglobulin G (IgG), Alexa Fluor® 594 goat anti-chicken IgG, Alexa Fluor® 594 goat anti-rabbit IgG (Life Technologies, Carlsbad, CA, USA) and biotin-conjugated goat anti-rabbit IgG (Nichirei Biosciences Inc., Tokyo, Japan). The nuclei were counterstained with DAPI (4′,6-diamidino-2-phenylindole), which was present in the mounting medium (Vectashield, Vector Laboratories, Burlingame, CA, USA) or Methyl Green (Merck Millipore, Darmstadt, Hessen, Germany). Specimens treated with biotin-conjugated secondary antibody were sensitized using streptavidin peroxidase (Vector Laboratories) and visualized using a diaminobenzidine (DAB) kit (Nichirei Biosciences Inc., Tokyo, Japan). The specimens were observed using a fluorescence microscope (BZ9000; Keyence, Osaka, Japan).

### Quantitative real-time PCR (qRT-PCR)

Total RNA from each organ, obtained from 7-week-old mice, was synthesized using the RNeasy® Mini Kit (Qiagen, Limburg, Holland). The mRNAs were reverse-transcribed into cDNAs using the SuperScript^™^ First-Strand Synthesis System (Invitrogen, Carlsbad, CA, USA). Gene expression was analyzed by qRT-PCR with a Bio-Rad C system (Bio-Rad, Hercules, CA, USA). The results were standardized to the housekeeping gene, β-actin. qRT-PCR was performed using an SYBR Supermix Kit (Bio-Rad) and the following conditions: 40 cycles at 95 °C for 15 sec and 60 °C for 45 sec. The melting curve data were collected to check the PCR specificity. Each cDNA sample was analyzed in triplicate. The threshold cycle (CT) was defined as the fractional cycle number. The gene expression value (relative mRNA level) was expressed relative to that of an internal reference gene (*actin*) that provides a normalization factor for the amount of RNA isolated from a specimen: ΔCt = Ct_interest_ − Ct_actin_. The Δ(ΔCt) method, i.e. Δ(ΔCt) = ΔCt_mutant_ − ΔCt_control_, was used to calculate the fold change, i.e. 2^− Δ(ΔCt)^, for each sample. All data are shown as the mean ± standard deviation (S.D.). The primers used were as follows: TRPM7 forward primer, AAAATCTATCGTTCAATGGC, reverse primer, CTTCCATGGGGATCTCTCTTCTG; TRPM6 forward primer, GGAGTAATTCCACCTGCTTG, reverse primer, GCGCTGTAGAACTCGTAGAC; β-actin forward primer, CTTTGCAGCCTCCTTCGTTGC, reverse primer, CCTTCTGACCCATTCCCACC.

### Western blot analysis

The first molars of 2-day postnatal mice were lysed in RIPA buffer (Wako Pure Chemical Industries Ltd., Osaka, Japan) supplemented with a protease inhibitor (Roche, Basel, Switzerland) and a phosphatase inhibitor (Roche). Total protein was separated by 12.5% polyacrylamide precast gel (Wako Pure Chemical Industries Ltd) and transferred to PVDF membranes (Millipore, Darmstadt, Hessen, Germany). The membranes were immunoblotted separately with mouse monoclonal anti-β-actin (Sigma-Aldrich, St. Louis, MO, USA), rabbit monoclonal anti-phospho-CREB (Ser133) or anti-CREB (Cell Signaling Technology) antibodies. The immunoblots were then incubated with peroxidase-conjugated secondary antibodies directed against mouse IgG (Sigma-Aldrich) or rabbit IgG (Bio-Rad, Hercules, CA, USA), treated with Amersham ECL Western Blotting Detection Reagent (GE Healthcare, Fairfield, CT, USA) and developed on X-ray film to detect protein expression levels.

### Co-immunoprecipitation experiments

A mouse inner enamel epithelial cell line derived from the apical bud of a mouse incisor (mHAT9d) was used. The cells were suspended in 1 mL of ice-cold RIPA buffer containing protease inhibitors (1 mM phenylmethane sulfonyl fluoride and 50 μg/mL protease inhibitor cocktail; Sigma-Aldrich). Cells were lysed by sonication, and the fragments were pelleted by centrifugation at 13000 rpm for 10 min at 4 °C. A 50% slurry of Protein A Sepharose 4 Fast Flow beads in RIPA buffer was prepared using the Immunoprecipitation (IP) Starter Pack (Amersham Biosciences, Piscataway, NJ, USA), as described in the manufacturer’s instructions. Supernatant from pelleted cell lysate (500 μL) was incubated with antibodies against TRPM7 (Scrum, Tokyo, Japan) and CREB overnight at 4 °C. To collect antibody-protein complexes, 50 μL of a 50% slurry of Protein A Sepharose was added to 500 μL of cell lysate and mixed for 1 h. IP beads were then collected by centrifugation at 1000 rpm for 2 min at 4 °C. The supernatant was discarded, and the beads were washed twice with 1 mL RIPA buffer and once with 50 mM ethylenediaminetetraacetic acid. Thereafter, IP beads were processed by SDS-PAGE. Proteins in the electrophoresed gel were blotted onto the Immobilon-P Transfer Membrane (Millipore) and detected by Western blotting with use of antibodies against Smad1/5, p38, CREB and TRPM7 proteins (Cell Signaling Technology). Proteins bound to the respective antibodies were visualized using an electrochemiluminescence detection system (Amersham Biosciences).

### Electrophysiologic measurements

The lower incisors from heterozygous control and TRPM7 KR mice at P5 were used. The lower incisor was dissected from the mandible and incubated with collagenase for 1 h at 4 °C to isolate the enamel layer from the mesenchyme. Single cells were isolated from the epithelial layer with trypsin solution and maintained in Dulbecco’s Modified Eagle’s Medium (D-MEM/Ham’s F-12; Wako Pure Chemical Industries Ltd.) containing 5% heat-inactivated fetal bovine serum (Mediatech Inc., Corning Life Sciences, Manassas, VA, USA) and 1% penicillin/streptomycin at 37 °C in a humidified atmosphere of 95% air/ 5% CO_2_.

The whole-cell configuration patch-clamp technique was used to record TRPM7 channel activity in primary inner enamel epithelium-lineage cells. A coverslip with adherent cells was placed in a recording chamber mounted on an inverted microscope (TMD300, Nikon, Tokyo, Japan) and continuously superfused (1 mL/min) with modified physiologic salt solution. For measurement of MIC currents (TRPM7-like currents), cells were dialyzed with Mg^2+^-free patch pipette solution (140 mM CsCH_3_SO_3_ [sodium methanesulfonate], 8 mM NaCl, 4 mM CaCl_2_, 10 mM EGTA and 10 mM HEPES, adjusted to pH 7.3 with CsOH) and superfused with Mg^2+^-free extracellular solution (140 mM NaCH_3_SO_3_, 6 mM CsCl, 1.5 mM CaCl_2_, 10 mM glucose and 10 mM N-2-hydroxyethyl-piperazine-N′-2-ethanesulfonic acid [HEPES], adjusted to pH 7.3 with tris-(hydroxymethyl)-aminomethane [Tris]). The Mg^2+^-dependence of the current was examined by altering the extracellular and intracellular Mg^2+^ concentrations (through addition of MgCl_2_) while keeping the free Ca^2+^ concentration unchanged. In some experiments, 2-APB (500 μM) or FTY720 (10 μM), known inhibitors of TRPM7 channel activity, were added to the extracellular bathing solution. 2-APB has been shown to inhibit native and overexpressed TRPM7 channels with IC50 in the 70~170 μM. As the preliminary experiment, we also confirmed that similar concentration range, 50 and 100 μM of 2-APB inhibited Mg^2+^ -inhibited cation (MIC) currents in inner enamel epithelium-lineage cells. However, highest concentration of 500 μM 2-APB caused significant inhibition of the MIC currents with a rapid onset as compared to the lower them. The previous articles have been also shown the effective concentration including 500 μM 2-APB^39,40^. The free Mg^2+^ and Ca^2+^ concentrations were estimated using WebMaxC software (http://web.stanford.edu/~cpatton/webmaxc/webmaxcS.htm). Recordings were made and digitized using an Axopatch 200 A amplifier and pCLAMP 10.2 software (Axon Instruments, Foster City, CA, USA). All electrophysiology experiments were performed at 26–27 °C.

### Statistical analysis

All data are shown as the mean ± S.D. Statistical comparisons were made using the Mann-Whitney U-test. A *P* value < 0.05 was considered statistically significant.

## Electronic supplementary material

Supplementary information
