# Supplementary material for: The crucial role of the TRPM7 kinase domain in the early stage of amelogenesis
Source: Sci Rep. 2017 Dec 22;7:18099. doi: 10.1038/s41598-017-18291-0 (PMC5741708; doi:10.1038/s41598-017-18291-0)
Supplement: Supplementary file 1 — Supplementary information [file 41598_2017_18291_MOESM1_ESM.pdf]

Supplementary Information for:

The crucial role of the TRPM7 kinase domain in the early stage of amelogenesis

Kayoko Ogata[1,2,¶] , Tomoyuki Tsumuraya[3,¶] , Kyoko Oka[2,*] , Masashi Shin[1], Fujio Okamoto[1], Hiroshi Kajiya[1], Chiaki Katagiri[3], Masao Ozaki[2], Masayuki Matsushita[3], Koji Okabe[1]

[1] Section of Cellular Physiology, Department of Physiological Sciences and Molecular Biology, Fukuoka Dental College

[2] Section of Pediatric Dentistry, Department of Oral Growth and Development, Fukuoka Dental College

[3] Department of Molecular and Cellular Physiology, Graduate School of Medicine, University of the Ryukyus

Supplementary Information 1
Full length blots cropped for representative figures.

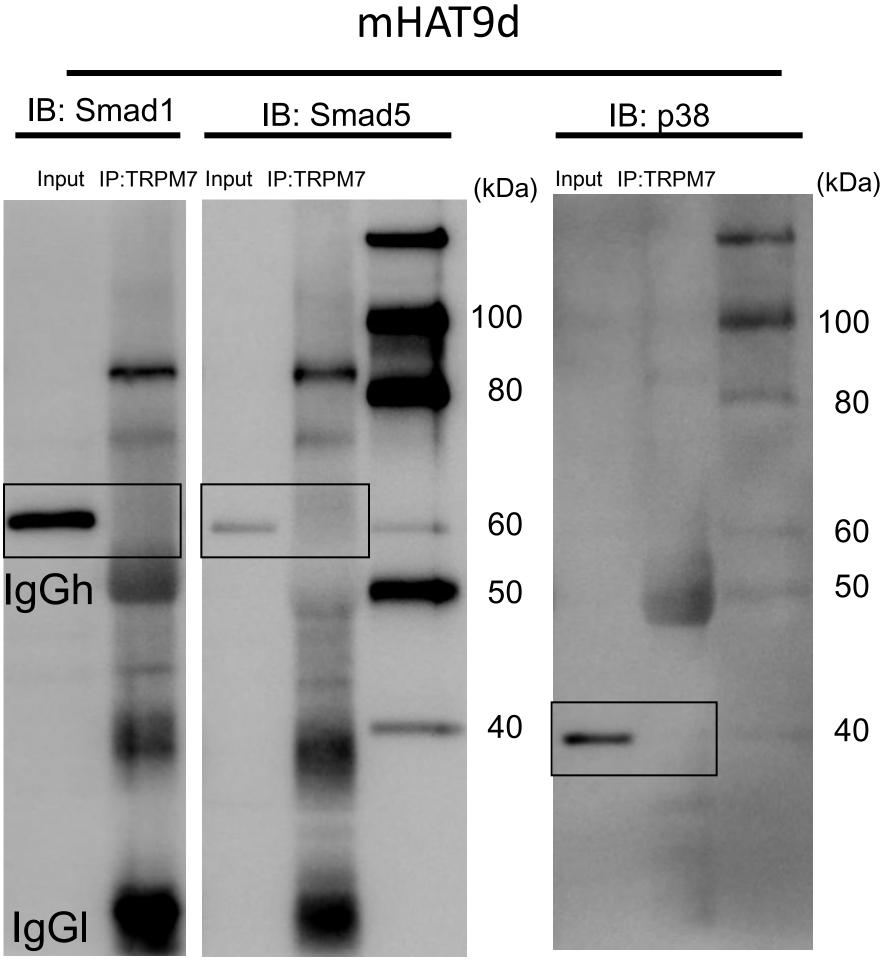

SI-1. Full length immunoblots shown in Figure 6S
Boxes indicate lanes which was used in Figure 6S.

Supplementary Information 2
Full length blots cropped for representative figures.

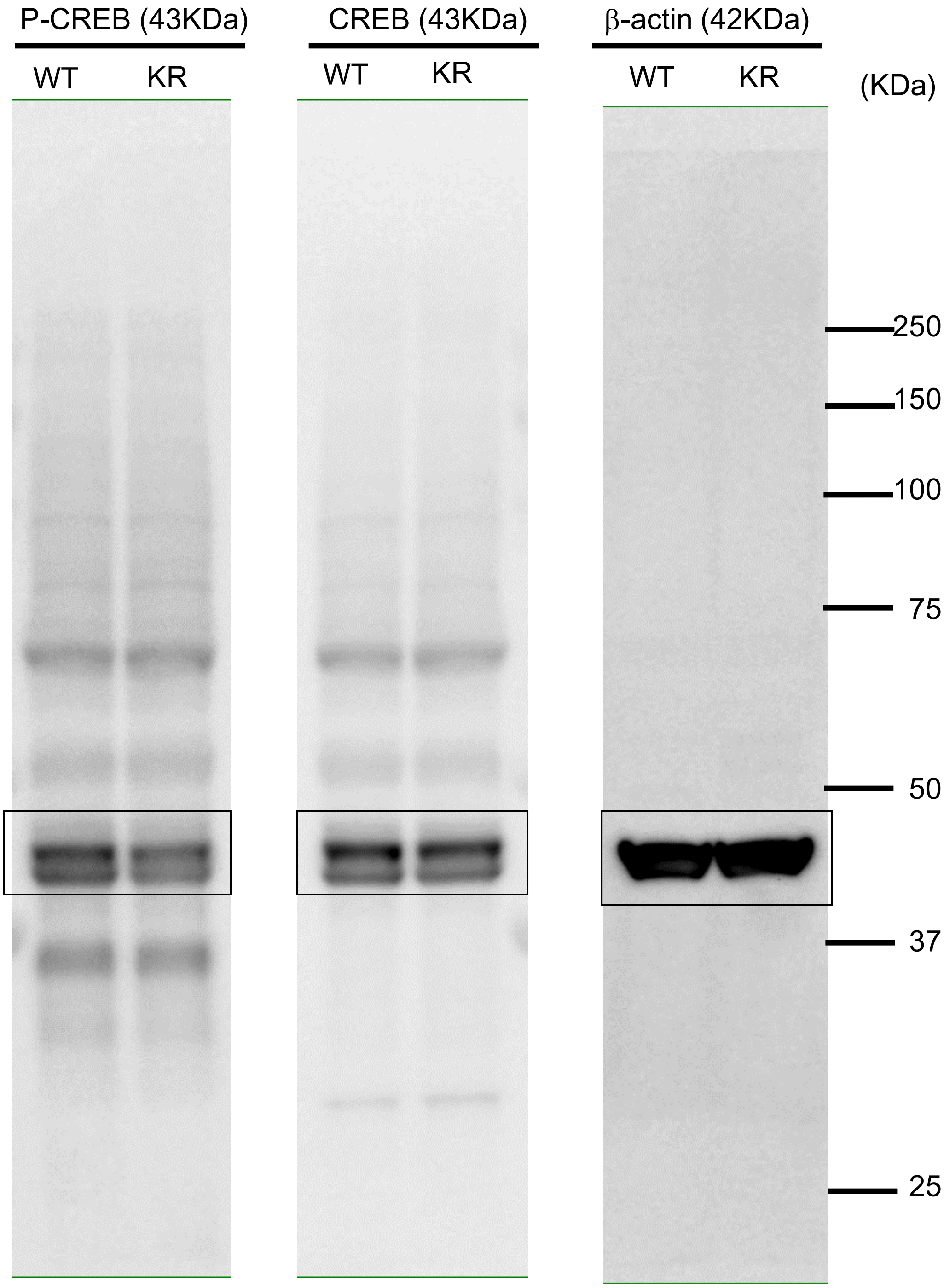

SI-2. Full length immunoblots shown in Figure 7M
Boxes indicate lanes which was used in Figure 7M.

Supplementary Information 3
Full length blots cropped for representative figures.

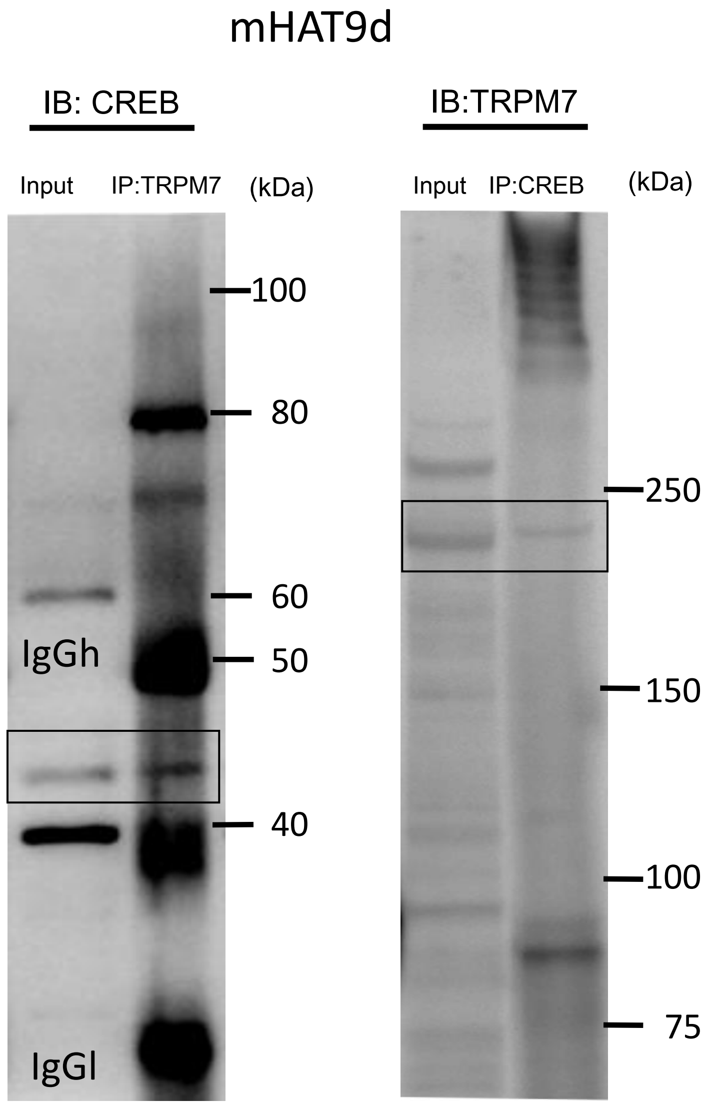

SI-3. Full length immunoblots shown in Figure 7N
Boxes indicate lanes which was used in Figure 7N.
